# The TLR4 Agonist Vaccine Adjuvant, GLA-SE, Requires Canonical and Atypical Mechanisms of Action for T_H_1 Induction

**DOI:** 10.1371/journal.pone.0146372

**Published:** 2016-01-05

**Authors:** Natasha Dubois Cauwelaert, Anthony L. Desbien, Thomas E. Hudson, Samuel O. Pine, Steven G. Reed, Rhea N. Coler, Mark T. Orr

**Affiliations:** 1 Infectious Disease Research Institute, Seattle, Washington, United States of America; 2 Department of Global Health, University of Washington, Seattle, Washington, United States of America; Imperial College London, UNITED KINGDOM

## Abstract

The Toll-like receptor 4 agonist glucopyranosyl lipid adjuvant formulated in a stable emulsion (GLA-SE) promotes strong T_H_1 and balanced IgG1/IgG2 responses to protein vaccine antigens. This enhanced immunity is sufficient to provide protection against many diseases including tuberculosis and leishmaniasis. To better characterize the adjuvant action it is important to understand how the different cytokines and transcription factors contribute to the initiation of immunity. In the present study using T-bet^-/-^ and IL-12^-/-^ mice and a blocking anti-IFNαR1 monoclonal antibody, we define mechanisms of adjuvant activity of GLA-SE. In accordance with previous studies of TLR4 agonist based adjuvants, we found that T_H_1 induction via GLA-SE was completely dependent upon T-bet, a key transcription factor for IFNγ production and T_H_1 differentiation. Consistent with this, deficiency of IL-12, a cytokine canonical to T_H_1 induction, ablated T_H_1 induction via GLA-SE. Finally we demonstrate that the innate immune response to GLA-SE, including rapid IFNγ production by memory CD8+ T cells and NK cells, was contingent on type I interferon, a cytokine group whose association with T_H_1 induction is contextual, and that they contributed to the adjuvant activity of GLA-SE.

## Introduction

Subunit vaccines, in combination with adjuvants to enhance the immune response to the target antigens, represent a significant advance in the development of better defined, easier to produce and safer vaccines. Importantly an optimal adjuvant should induce a targeted innate response to tailor the desired adaptive response needed for vaccine efficacy. To that end we have developed a number of Toll-like receptor (TLR) agonists containing adjuvants that promote T_H_1 T cell responses to vaccine antigens. The most notable of these is the GLA-SE adjuvant which contains a synthetic TLR4 agonist, Glucopyranosyl Lipid Adjuvant (GLA), formulated in a stable nano-emulsion of squalene oil-in-water (SE) [1, 2]. GLA-SE drives strong T_H_1 responses to a variety of antigens that are protective against intracellular infections [2–7]. In combination with the tuberculosis vaccine fusion protein antigen ID93, GLA-SE induces a poly-functional T_H_1 response characterized by CD4 T cells producing CD154, IFNγ, TNF, GM-CSF, and IL-2, as well as a humoral response skewed towards IgG2c class-switching [8–10]. In order to better understand the mechanism of action of adjuvants it is important to define the role of different cytokines and transcription factors in initiating the immune response from the naive polyclonal repertoire.

The differentiation of CD4 T cells into T_H_1 effectors is orchestrated by the transcription factor T-bet [11]. This differentiation occurs in two steps: first, during the initial polarization phase, simultaneous signaling via the T cell antigen receptor TCR and the IFNγ receptor of the naïve T cell results in T-bet expression which enables IL-12 receptor expression, and subsequently a second wave of T-bet expression is induced by IL-12 signaling in the absence of TCR stimulation [12–15]. T-bet induction and IL-12 production are therefore likely necessary for the potent T_H_1 response induction to vaccination with GLA-SE.

Type I interferons (IFNα and IFNβ) induce an antiviral state in most nucleated cells, providing protection against infection [16, 17]. Furthermore, type I IFN can shape the adaptive responses to infection (reviewed in [18]). These cytokines signal via the heterodimeric IFNαR1/2 receptor and act on both antigen presenting cells (APC) and lymphocytes to enhance maturation, proliferation and survival to a variety of stimuli [19]. In the present study, using T-bet^-/-^ and IL-12^-/-^ mice and IFNαR1 antibody blockade we demonstrate that T-bet induction, IL-12 production and IFNαR1 signaling are necessary for the adjuvant activity of GLA-SE and that IFNαR1 signaling is also crucial for the early innate response initiation to this adjuvant.

## Results

### GLA-SE adjuvant activity is dependent on T-bet expression and IL-12 production

**Fig 1:**
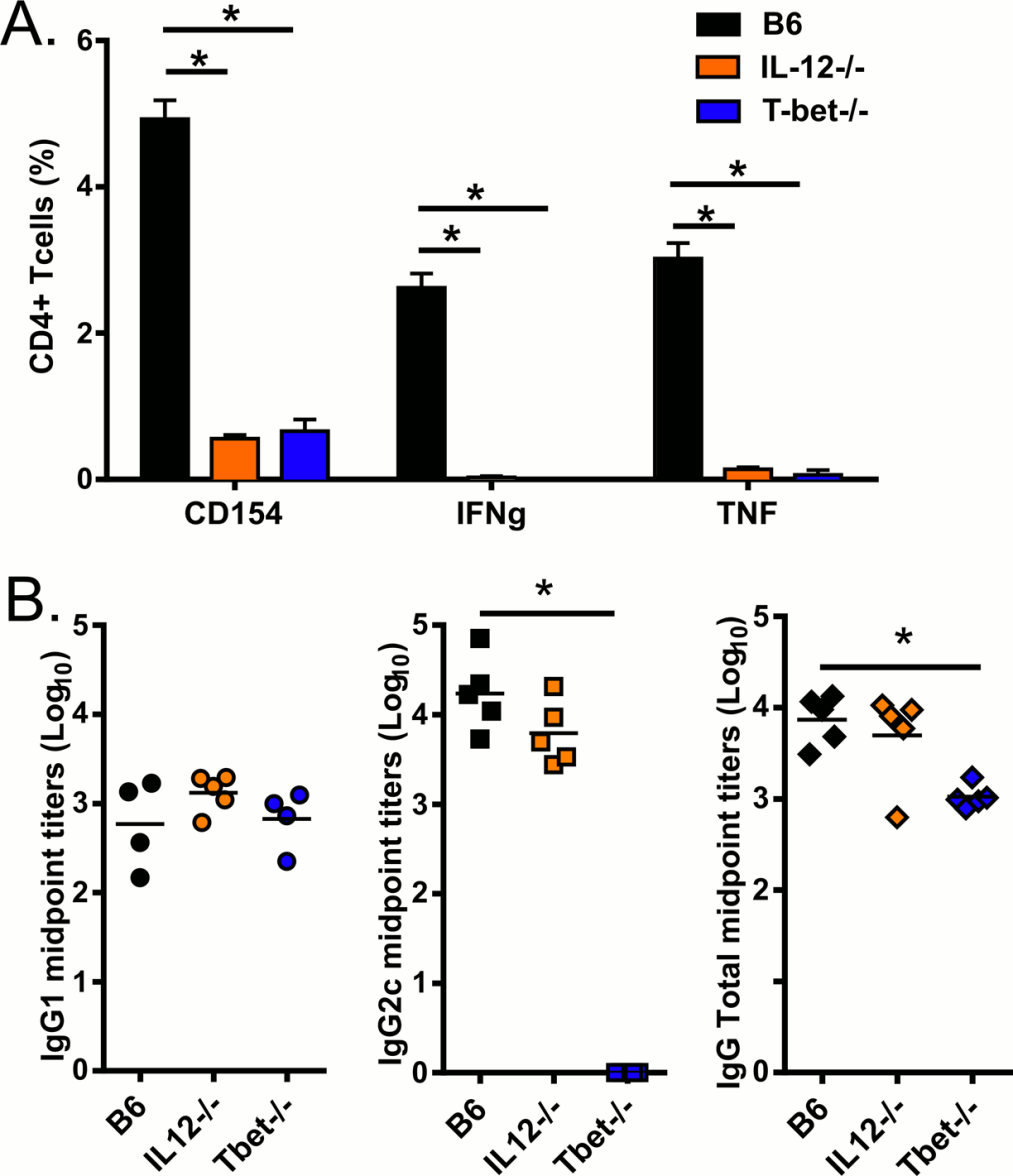
ID93+GLA-SE T_H_1 response is dependent on T-bet and IL-12 whereas antibody production is only dependent on T-bet. B6, IL12^-/-^ and T-bet^-/-^ mice were immunized with ID93+GLA-SE. (A)One week after boost, splenocytes were isolated and stimulated with ID93. CD4 T cells were analyzed for the production of CD154, IFN-γ, and TNF. (B) Sera were collected three weeks after the first immunization and serially diluted to assess levels of anti-ID93 IgG1, IgG2c and IgG Total. Data are shown as mean ± SEM of N = 4–5 animal/group and are from one experiment representative of two experiments performed. Statistics by two-way ANOVA with Dunnett’s correction for multiple comparison test within IgG and cytokine groups versus B6; *p≤0.05.

GLA-SE, a synthetic TLR4 agonist formulated in a stable nano-emulsion of squalene oil induces a strong T_H_1 response to vaccines antigens that otherwise elicit minimal cellular immune responses with a T_H_2 bias [1, 9, 10, 20]. IL-12 is important for T_H_1 induction with LPS, another TLR4 agonist, and monophosphorylated lipid a (MPLA), a detoxified derivative of LPS [21, 22]. Mouse and human dendritic cells stimulated with GLA produce IL-12 in a MyD88 and TRIF dependent manner [2, 9]. To determine whether IL-12 production and/or T-bet expression are important for GLA-SE driven antibody and CD4 T cell responses, we immunized wild type (B6) or IL-12 or T-bet deficient mice with GLA-SE and the recombinant antigen ID93. Both T-bet and IL-12 were essential for T_H_1 induction as indicated by CD4+ T cells production of CD154, IFN-γ, or TNF upon stimulation with ID93 (Fig 1A). Following immunization with ID93+GLA-SE induction of a strong IgG2c skewed response to ID93 was completely dependent on T-bet expression, but, surprisingly, not on IL-12 (Fig 1B). These data suggest discordance between an IL-12 dependent induction of IFNγ- producing CD4 T cells and an IL-12 independent class-switching to IgG2c, with T-bet being essential for both.

### GLA-SE induces IFNα *in vivo*

**Fig 2:**
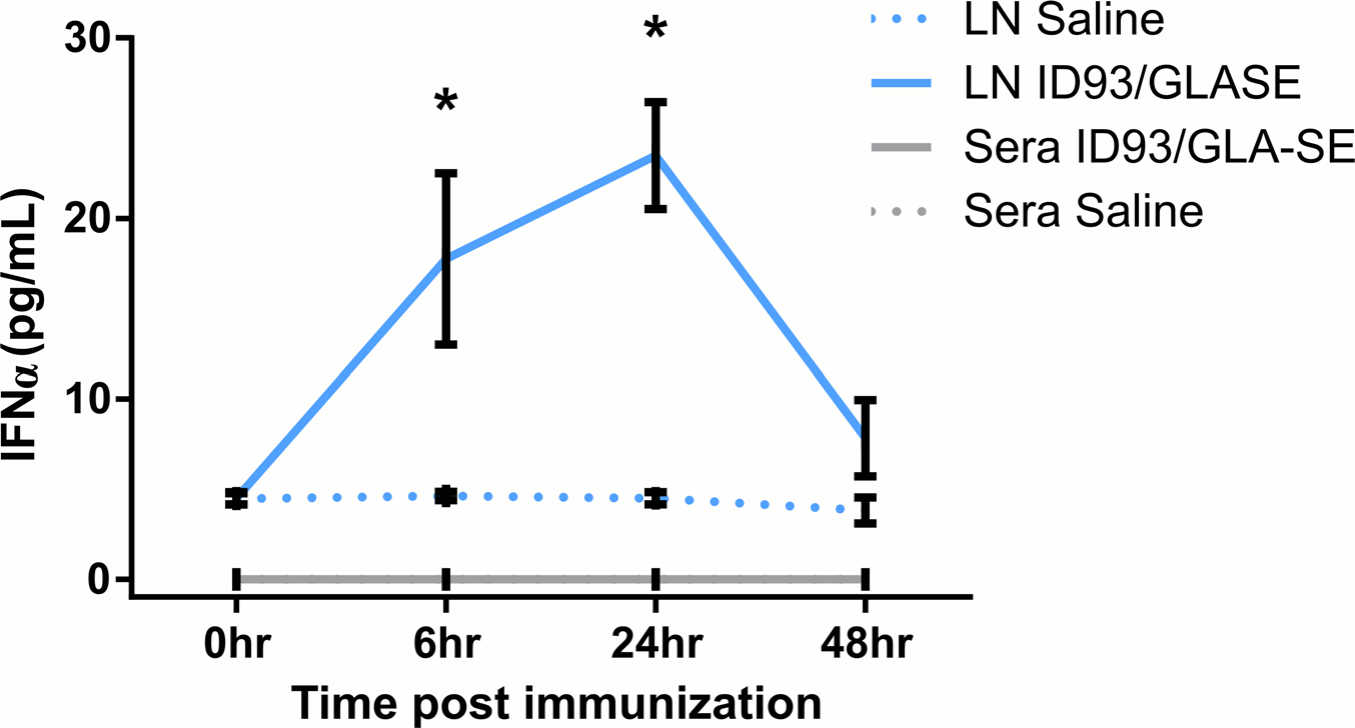
IFNα is produced early after GLA-SE immunization. B6 mice were immunized with saline or ID93+GLA-SE. Sera and draining inguinal LNs were collected0, 6, 24, or 48 hours later and analyzed for IFNα protein expression by ELISA. Data are shown as mean ± SEM and are the combined results of two independent experiments with similar results with 3 or 4 mice/group. Statistics by unpaired t test; *p≤0.05 compared to Saline group.

GLA induces transcription of type I IFN by human dendritic cells *in vitro*. *In vivo* induction of CD69 on T cells by GLA-SE is partially dependent on cell-intrinsic expression of type I IFN receptor [2, 23]. Type I IFN are TRIF-dependent cytokines produced by TLR4 stimulation with GLA or similar agonists [2, 24, 25]. MyD88 and TRIF are both necessary for GLA-SE adjuvant activity [9]; suggesting a role for type I IFN in the T_H_1 differentiation induction by GLA-SE. To determine whether GLA-SE induced type I IFN *in vivo*, we measured IFNα levels in the draining lymph node (LN) and sera shortly after immunization with GLA-SE. IFNα levels in the draining LN peaked between 6 and 24 hours and returned to almost undetectable levels at 48 hours (Fig 2). No IFNα was detected in the serum indicating that the response was highly localized to draining LNs (Fig 2).

### Type I IFN receptor signaling is critical during the innate response to GLA-SE

**Fig 3:**
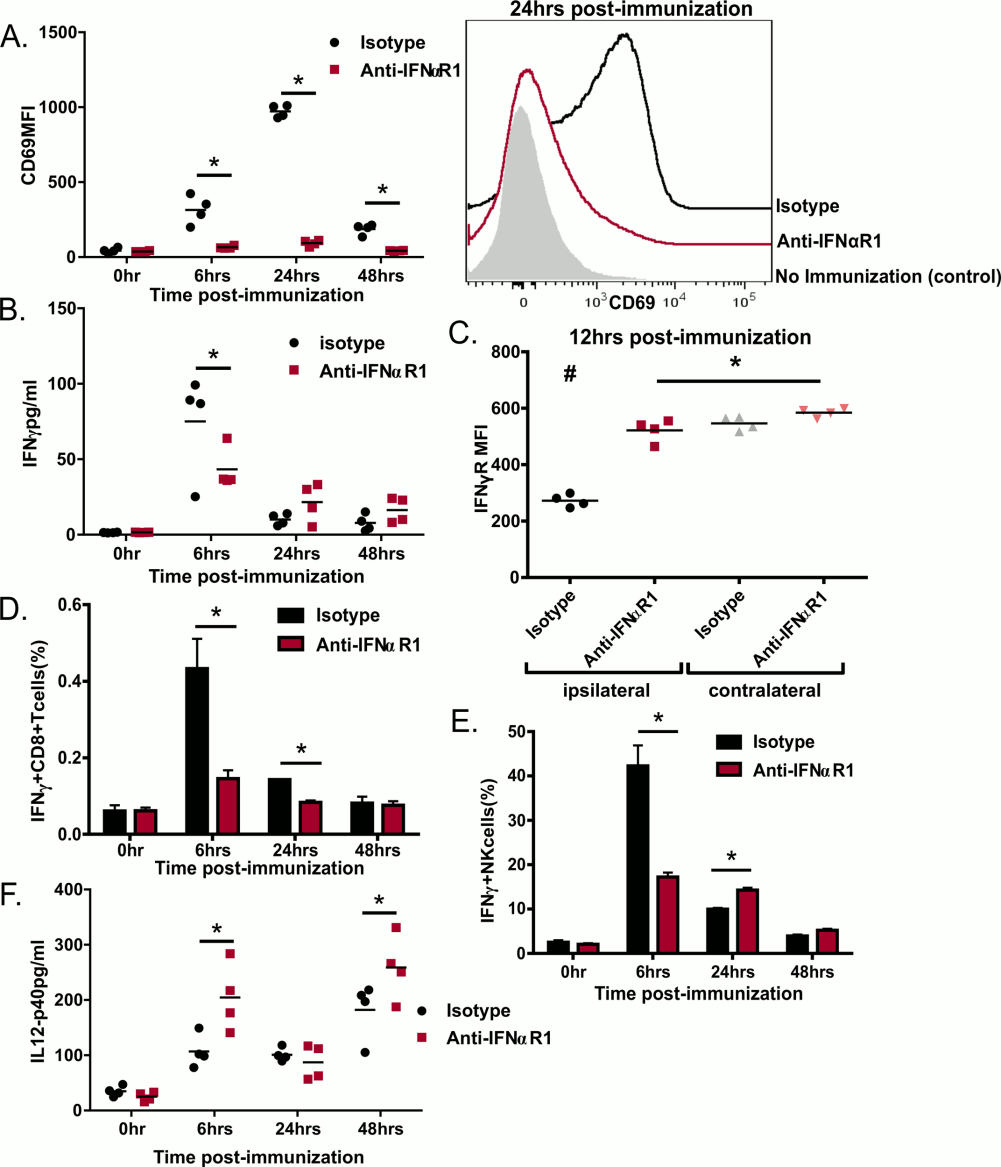
IFNαR1 signaling is essential for lymphocyte activation and IFNγ production upon immunization with ID93+GLA-SE. B6 mice were treated with IFNαR1 antibody or its isotype and immunized with ID93+GLA-SE. Innate responses were assessed in the ipsilateral draining LN or in the contralateral LN by flow cytometry on LN cells or ELISA on LN supernatant The results are shown for the ipsilateral LN when not stated otherwise. (A) MFI and representative histograms of CD69 staining (B) IFNγ production assessed by ELISA, (C) IFNγR occupancy as indicated by decreased MFI of IFNγR staining with the monoclonal antibody GR-20 at 12 hrs after immunization. (D) IFNγ staining on CD8+ T cells. (E) IFNγ staining on NK cells. (F) IL-12 production assessed by ELISA. Data are shown as mean ± SEM of N = 4 animal/group and are from one experiment. Statistics by one-way ANOVA, *p≤0.05 compare to anti-IFNαR1 treated animals, #p≤0.05 compare to all the other groups.

Type I IFN drive expression of the cell surface activation marker CD69 which in turn facilitates trapping of naïve lymphocytes in the draining LN with antigen-loaded APCs [26]. Immunization with GLA-SE induces CD69 on lymphocytes, independent of antigen-specificity and this induction is partially dependent on cell intrinsic expression of type I IFN receptor as well as innate phase production of IFNγ. Similar to our previous findings [23] immunization with GLA-SE drove a transient expression of CD69 on the total lymphocyte population in the draining LN, which was dependent on type I IFN signaling (Fig 3A). GLA-SE immunization also produced a strong burst of IFNγ in the draining LN 6hr after immunization which returned to baseline by 24 hours (Fig 3B). This was also dependent on type I IFN as blocking the IFNαR1 ablated most of the IFNγ production (Fig 3B).

The occupancy of IFN-γ receptor, and therefore the amount of IFNγ present on the surface of cells, can be determined by staining with the empty-IFNγ receptor-specific antibody clone GR-20 [14]. Correlating with our IFNγ production results, 12hrs after immunization the empty-IFNγ receptor staining on lymphocytes from draining LNs was substantially reduced in mice treated with GLA-SE compared with contralateral LNs. IFNαR1 blockade preserved empty-IFNγ receptor staining confirming that the early IFNγ burst depends on IFNαR1 signaling (Fig 3C). This early burst of IFNγ at 6 hr was produced by both memory CD8 T cells and NK cells (Fig 3D and 3E). This is likely driven by IL-18 expression which is induced by GLA-SE and necessary for the adjuvanticity of GLA-SE [23]. IFNγ production by both cell types was strongly reduced in mice that were previously treated with anti-IFNαR1 (Fig 3D and 3E). These results indicate that type I IFN, in sequential manner, was necessary for the early innate production of IFNγ by CD8+ T cells and NK cells in response to GLA-SE.

Concurrent with the early IFNγ production there is an increase in IL-12 production in the draining LN following GLA-SE immunization as early as 6 hours post immunization which is sustained for at least 2 days and is critical for the adjuvant activity of GLA-SE (Figs 1 and 3F). Surprisingly IFNαR1 blockade augmented the levels of IL-12 in the draining LN despite IFNγ levels being lower in the treated animals at the same time-points (Fig 3F). These results suggest that type I IFN production inhibits early IL-12 production, which is supported by prior reports [27–30]. Overall the innate activity of GLA-SE is strongly dependent on type I IFN which is necessary for lymphocyte activation, controls IL-12p40 production and promotes early IFNγ production, a feature associated with T-bet expression in T cells, and subsequently T_H_1 induction [31].

### GLA-SE T_H_1 induction and T_H_2 counter-regulation is partially dependent on type I IFN production

**Fig 4:**
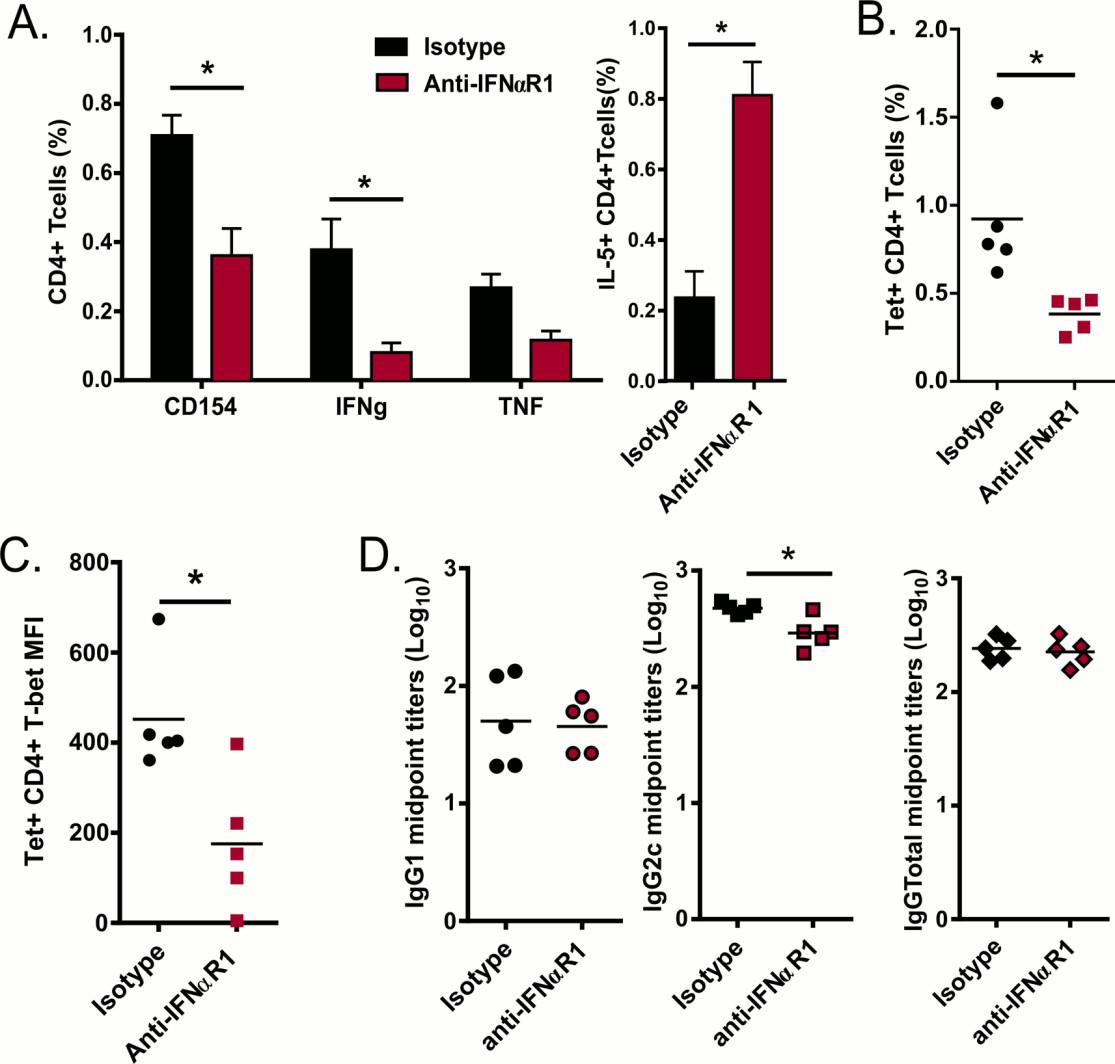
IFNαR1 signaling contributes to T_H_1 skewing. B6 mice were treated with IFNαR1 antibody or its isotype and immunized with ID93+GLA-SE and responses were analyzed one week after prime. (A) ID93 stimulated splenocytes were analyzed for the production of CD154, IFNγ, TNF and IL-5 by CD4 T cells. (B) One week after prime CD4 T cells were isolated and stained with an I-A^b^ tetramer presenting the dominant epitope for Rv3619 and analyzed for T-bet induction. (C) Sera were collected one week after prime and serially diluted to assess levels of anti-ID93 IgG1 and IgG2c. Data are shown as mean ± SEM of N = 5 animal/group and are from one experiment representative of two experiments performed except for IL-5 and T-bet levels measurement which were only done once. Statistics by simple or multiple t test corrected for multiple comparisons using the Holm-Sidak method between IgG or cytokine groups; *p≤0.05

To further investigate the role of type I IFN in the adaptive response elicited by immunization with GLA-SE and ID93, we blocked IFNαR1 and examined the cellular and humoral responses one week after immunization. IFNαR1 blockade reduced the frequency of antigen-specific CD4 T cells as measured by CD154 expression upon stimulation (Fig 4A). Further, T_H_1 responses were reduced and T_H_2 responses enhanced, as measured by antigen induced production of IFNγ, TNF and IL-5 (Fig 4A). In parallel, samples were stained for ID93-specific CD4 T cells via peptide-MCHII tetramer (Tet) staining. Consistent with CD154 expression upon stimulation there was a significant reduction in the frequency of Tet+ CD4 T cells from the IFNαR1 blockade group (Fig 4B). Congruent with the reduction in IFNγ observed after antigen stimulation, the remaining Tet+ T cells from the IFNαR1 blockade group had lower levels of T-bet staining (Fig 4C). The reduction in T_H_1 skewing correlated with modestly decreased isotype switching to IgG2c in ID93-specific antibody titers (Fig 4D). Taken together these results suggest that GLA-SE induced type I IFN promote T_H_1 responses by enhancing CD69 expression and increasing innate IFNγ production.

## Discussion

GLA-SE is a clinical stage vaccine adjuvant that robustly augments T_H_1 immunity yet its mode of action is still incompletely understood. In this study we assessed the contribution of T-bet, IL-12 and IFNαR1 signaling to the adjuvant activity of GLA-SE. We show that T-bet is required for IgG2c isotype skewing as well as T_H_1 induction by GLA-SE. Thereupon we sought to investigate the role of the pro-inflammatory cytokine IL-12 and, surprisingly, found that IgG2c class-switching was not impaired even though production of IFN-γ secreting CD4 T_H_1 cells was ablated. Finally, our results suggest that type I IFN, which are induced early after immunization with GLA-SE, are necessary for an optimal innate response including rapid lymphocyte activation and IFNγ expression by NK cells and memory CD8 T cells and, consequently, the induction of T_H_1 immunity.

IL-12 is a key factor that drives T_H_1 responses and IFNγ production [23, 32–35]. However, the relevance of IL-12 *in vivo* depends on the nature of the infection or immunization. In IL-12-deficient mice, T_H_1-type responses were generated upon immunization with inactivated MHV, demonstrating the existence of an IL-12 independent T_H_1 induction that has been since then described in several models of viral infection and immunization [36–41]. Conversely, IL-12 has been shown to be important for T_H_1 induction by the TLR4 agonists LPS and MPL, although antibody production was not evaluated in those papers [21, 22]. Using IL-12^-/-^ and T-bet^-/-^ mice, we demonstrated that IL-12 is needed for the CD4 T_H_1 induction by GLA-SE but not for IgG2c skewing, whereas T-bet is necessary for both. Since B-cell intrinsic T-bet has been shown to mediate early IgG2c production [42] further experiments will be needed to determine if T-bet expression upon ID93-GLA-SE vaccination is required in the CD4 T cells to promote IgG2c skewing. Furthermore we have previously shown that MyD88 and TRIF are both necessary for GLA-SE T_H_1 skewing capacity as well as IL-12 production by BMDC, DC and macrophages [9]. This suggests that IL-12 production might be a necessary point of intersection for the previously demonstrated MyD88- and TRIF-dependent induction of T_H_1 responses by ID93+GLA-SE immunization. Moreover IL-18 has been shown to synergize with IL-12 to induce IFNγ in T cells [34, 35]. IL-18 is produced very early upon immunization with GLA-SE and IL-18R^-/-^ mice have a reduced antigen-specific T_H_1 response [23]. We hypothesize that this IL-18/IL-12 synergy is required for GLA-SE-induced T_H_1 responses.

Type I IFN links the innate and adaptive immune responses. They cause APC maturation *in vitro* while inhibiting secretion of T_H_2 cytokines leading to IFNγ producing T cells [43–45]. Type I IFN inhibit the death of CD4 activated T cells and their key role in priming adaptive T cells has been demonstrated in a wide variety of immunization schemes [46, 47]. Recombinant type I IFN given *in vivo* has direct adjuvant activity, enhancing both antibody and T cell responses [48, 49]. TLR agonist and non-TLR agonist require type I IFN activity to exert their adjuvant activities [18, 48, 50]. In some cases this may be due to IFNαR signaling altering the expression of some TLRs [43]. Furthermore Longhi *et al* observed an IL-12 independent impairment of T_H_1 responses to poly (I:C) when blocking IFNαR1 and in IFNαR^-/-^ mice [39]. Thus it has been hypothesized that robust type I IFN induction may be necessary for effective adjuvant activity [51]. However other adjuvants including MF59 and Pam3CSK (TLR2) do not elicit type I IFN and do not require IFNαR signaling to enhance antibody responses [50].

Our results show that that type I IFN is critical for the adjuvanticity of GLA-SE. Type I IFN was required for maximal response to this adjuvant illustrated by defects in early innate responses as indicated by reduced CD69 expression and defects in innate IFNγ production by CD8+ T cells and NK cells. CD69 expression inhibits lymphocytes egress from the LN which is a mechanism to retain useful clonal specificities in the activated LN [26, 52]. Early IFNγ produced by innate cells upon immunization with GLA-SE and other adjuvants is critical for CD4 T cells T_H_1 polarization in the LN [13, 15, 23]. IFNαR1 blockade caused multiple defects in T_H_1 adaptive responses including: (1) reduction in the frequency of antigen-specific cells determined by CD154 production or tetramer staining, (2) reduction in T_H_1 skewing measured by IFNγ or TNF production and lower amounts of the T_H_1 controlling transcription factor Tbet in the remaining tetramer positive cells, and (3) enhanced frequencies of CD4 T cells that produced IL-5, a hallmark of T_H_2 differentiation.

In some settings type I IFN is necessary for IL-12 production by murine and human dendritic cells [53, 54] but they also have been shown to decrease IL-12 production in other studies [27–30]. IFNαR1 blockade increases IL-12 production early after immunization with GLA-SE but diminished IFNγ production at the same time indicating a complex regulation of IFNγ upon vaccination. We hypothesize that type I IFN driven IFNγ is necessary to increase expression of the IL-12R on CD4 T cells, thus making them responsive to IL-12. Alternatively production of the IL-12p35 subunit to form active IL-12p70 may be dependent on IFNαR1 signaling. In addition to their role in stimulating APCs to enhance CD4 responses, type I IFN can also have a direct effect on T cells. CD8 T cells require direct type I IFN signals for their expansion in response to some infections [55]. Likewise direct type I IFN action on CD4 T cells is important for clonal expansion *in vivo* following LCMV, but inhibitory during Listeria infection [56] showing that the priming milieu determines the extent to which CD4 T cells are dependent on direct signal mediated by type I IFN. Conversely CD4 T cell responses are dependent on type I IFN in a cell extrinsic manner in response to polyIC:CD40 vaccination [57]. Interestingly deletion of IFNαR2 on T cells significantly diminished CD69 expression induced by GLA-SE at 18h after immunization suggesting a dependence on T cell intrinsic expression of the type I IFN receptor for GLA-SE innate response induction [23]. Based on our results we suggest a model where GLA-SE induces IFNα to trap cells in the LN by CD69 expression and to augment the early innate IFNγ production by CD8+ T cells and NK cells. In parallel GLA-SE induces production of IL-12 *in vivo* which synergizes with IFNα effects to promote T-bet expression and T_H_1 commitment. Understanding the mechanism by which adjuvants engage the immune responses is critically important for development of vaccines. Our results suggest that early type I and II IFNs are signatures of the optimal innate response to the GLA-SE adjuvant, predicting subsequent T_H_1 responses. Further research is warranted to determine if this early type I IFN induction can be used as an early gating strategy in developing new adjuvants or as a signature of adjuvanticity in human clinical trials.

## Materials and Methods

### Ethics statement

The study was conducted under protocols approved by the Infectious Disease Research Institute Institutional Animal Care and Use Committee.

### Animals

6–8 week old female C57BL/6 (B6), IL-12^-/-^ and T-bet^-/-^ mice were purchased from Jackson Lab and maintained in Specific Pathogen Free conditions. All animal study protocols were approved by the IDRI Institutional Animal Care and Use Committee (IACUC) and were performed according to IACUC regulations and guidelines.

### Immunizations and IFNαR1 blockade

Mice were immunized with 0.5 μg of ID93 recombinant protein [8] formulated in 5 μg of GLA (Avanti Polar Lipids) in IDRI’s stable emulsion (SE) [2] by intramuscular injection. For IFNαR1 blockade, 1 mg of anti-IFNαR1 antibody (BioXCell, clone MAR1-5A3) or its IgG1 isotype control (BioXCell, clone MOPC-21) in PBS were injected i.p. 6h prior to immunization and on day 3 after immunization.

### IFNα ELISA

At indicated times following immunization peripheral blood and inguinal LNs were collected and LNs were homogenized in 500μL PBS. Total production of IFNα was assessed using the Mouse IFNα ELISA kits (R&D Systems) according to the manufacturer’s instructions. Concentrations below the limit of detection were reported as 0.

### Antibody responses

Mouse sera were prepared by collection of retro-orbital blood into microtainer serum collection tubes (VWR International, West Chester, PA), followed by centrifugation at 10,000 rpm for 5 minutes. Each serum sample was then analyzed by antibody capture ELISA. Briefly, ELISA plates (Nunc, Rochester, NY) were coated with 2 μg/ml recombinant antigen ID93 in 0.1 M bicarbonate buffer and blocked with 1% BSA-PBS. Then, in consecutive order and following washes in PBS/Tween20, serially diluted serum samples, anti-mouse IgG1 or IgG2c-HRP (all Southern Biotech, Birmingham, AL) and ABTS-H_2_O_2_ (Kirkegaard and Perry Laboratories, Gaithersburg, MD) were added to the plates. Plates were analyzed at 405nm (EL_X_808, Bio-Tek Instruments Inc, Winooski, VT).

### Splenocytes recalls and intracellular cytokine staining

Splenocytes were isolated from three to five animals per treatment regimen. Red blood cells were lysed using Red Blood Cell Lysis Buffer (eBioscience) and resuspended in RPMI 1640, 10% FBS. Total viable cells were enumerated using ViaCount assay with a PCA system (Guava Technologies), plated at 2x10^6^ cells/well in 96-well plates and stimulated for 2 hours with media or ID93 (10 μg/mL) at 37°C. GolgiPlug (BD Biosciences) was added and the cells were incubated for an additional 8 hours at 37°C. Cells were washed and surface stained with fluorochrome labeled antibodies to CD4 (clone GK1.5) and CD8 (clone 53–6. 7) (BioLegend and eBioscience) in the presence of 20% normal mouse serum for 20 minutes at 4°C. Cells were washed and permeabilized with Cytofix/Cytoperm (BD Biosciences) for 20 minutes at room temperature. Cells were washed twice with Perm/Wash (BD Biosciences) and stained intracellularly with fluorochrome labeled antibodies to CD154 (clone MR1) IFN-γ (clone XMG-1.2), TNF (MP6-XT22) and IL-5 (TRFK5) (BioLegend and eBioscience) for 20 minutes at room temperature. Cells were washed and resuspended in PBS. Up to 10^6^ events were collected on a four laser LSRFortessa flow cytometer (BD Biosciences). Cells were gated as singlets > lymphocytes > CD4+ CD8- >cytokine positive. ID93-specific response frequencies were determined by subtracting the frequency of response positives of unstimulated cells from ID93 stimulated cells in matched samples.

For T-bet staining, CD4 T cells were stained with an I-A^b^ tetramer presenting the dominant epitope for Rv3619 [20] in the presence of 20% normal mouse serum for 1h at 37°C. APC labeled tetramers were provided by the NIH Tetramer Core Facility. Cells were washed and surface stained with antibodies to CD4 (clone GK1.5), CD8 (clone 53–6.7), CD19 (clone 1D3), Ly6G (clone 1A8), Ter119 (clone TER-119), F4/80 (clone BM8), CD11b (clone M1/70) and CD11c (clone N418) (Lin) (BioLegend and eBioscience). Cells were washed and permeabilized with Cytofix/Cytoperm (BD Biosciences) for 20 minutes at room temperature. Cells were washed twice with Perm/Wash (BD Biosciences) and stained intracellularly with fluorochrome labeled antibodies to T-bet (clone 4b10) overnight at 4°C. Cells were washed and resuspended in PBS. Up to 10^7^ events were collected on a four laser LSRFortessa flow cytometer (BD Biosciences). Cells were gated as singlets > lymphocytes > CD4+ Lin- >Tetramer+. Data were analyzed with FlowJo v9.8.

### LN cell staining and cytokine detection

Inguinal LN were collected in PBS 0.5%BSA with protease inhibitors and 10μg/mL BrefeldinA on ice and mechanically homogenized in PBS. Cells were surface stained with the labeled antibodies to IFNγR (clone GR20), CD69 (clone H1.2F3), CD8 (clone 53–6.7), CD90.2 (clone 30-H12) and NK1.1 (clone PK136) (BioLegend and eBioscience) in the presence of 20% normal mouse serum for 20 minutes at 4°C. Cells were washed twice with Perm/Wash (BD Biosciences) and stained intracellularly with fluorochrome labeled antibodies to IFNγ (clone XMG1.2) overnight at 4°C. Cells were washed twice with Perm/Wash (BD Biosciences) washed and resuspended in PBS. Up to 10^6^ events were collected on a four laser LSRFortessa flow cytometer (BD Biosciences). Lymphocytes were gated as singlets > lymphocytes (based on SSC x FSC) and CD69 and IFNγR MFI were assessed. CD8+ T cells were gated as singlets > lymphocytes > CD90.2+ CD8+ and NK cells were gated as singlets > lymphocytes CD8- NK1.1+. Data was analyzed with FlowJo v9.8.

### Statistical methods and figures

Statistical analyses and figures were performed using Prism software (GraphPad Software, Inc., La Jolla, CA). T-test were used when comparing one group against another and one-way or two ways ANOVA analyses were used when more than two groups were compared over. Non-normal data sets were log-transformed prior to analysis. Statistical significance was considered when the p-values were <0.05 and noted * or # when the group was statistically different to all the other groups.
